# Traditional potato tillage systems in the Peruvian Andes impact bacterial diversity, evenness, community composition, and functions in soil microbiomes

**DOI:** 10.1038/s41598-024-54652-2

**Published:** 2024-02-17

**Authors:** Aura L. García-Serquén, Lenin D. Chumbe-Nolasco, Acacio Aparecido Navarrete, R. Carolina Girón-Aguilar, Dina L. Gutiérrez-Reynoso

**Affiliations:** 1 Laboratorio de Biología Molecular y Genómica, Dirección de Recursos Genéticos y Biotecnología, Instituto Nacional de Innovación Agraria (INIA), Av. La Molina 1981, 15024 Lima, Peru; 2 Graduate Program in Environmental Sciences, Brazil University (UB), Estrada Projetada F1, Fazenda Santa Rita, Fernandópolis, São Paulo 15613-899 Brazil

**Keywords:** Soil microbiology, Microbial ecology

## Abstract

The soil microbiome, a crucial component of agricultural ecosystems, plays a pivotal role in crop production and ecosystem functioning. However, its response to traditional tillage systems in potato cultivation in the Peruvian highlands is still far from understood. Here, ecological and functional aspects of the bacterial community were analyzed based on soil samples from two traditional tillage systems: 'chiwa' (minimal tillage) and 'barbecho' (full tillage), in the Huanuco region of the Peruvian central Andes. Similar soil bacterial community composition was shown for minimal tillage system, but it was heterogeneous for full tillage system. This soil bacterial community composition under full tillage system may be attributed to stochastic, and a more dynamic environment within this tillage system. 'Chiwa' and 'barbecho' soils harbored distinct bacterial genera into their communities, indicating their potential as bioindicators of traditional tillage effects. Functional analysis revealed common metabolic pathways in both tillage systems, with differences in anaerobic pathways in 'chiwa' and more diverse pathways in 'barbecho'. These findings open the possibilities to explore microbial bioindicators for minimal and full tillage systems, which are in relationship with healthy soil, and they can be used to propose adequate tillage systems for the sowing of potatoes in Peru.

## Introduction

The soil microbiome plays an important role in food production and ecosystem functions, and recent studies have highlighted its relationship with human health^1^. In addition, soil microbiomes exhibit a great diversity of organisms that perform vital ecological functions, such as nitrogen and carbon cycles. These ecological roles of soil microbiomes maintain terrestrial ecosystems^2^ and plant productivity^3^, thereby mitigating the effects of climate change by increasing agroecosystem resilience^4^. Therefore, it is necessary to understand the composition and the functional profile of microbiome agroecosystems^5^.

**Figure 1:**
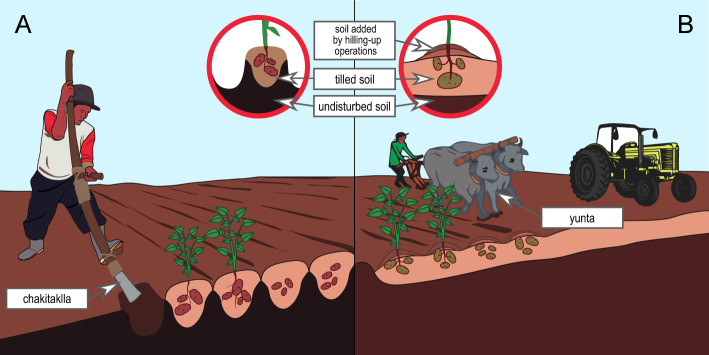
Traditional tillage systems. (**A**) Minimal tillage system ‘chiwa’ in which we can see the use of the ‘chakitaklla’ and the form of tillage on the soil. (**B**) Full tillage ‘barbecho’ in which we can see the use of yunta and tractor and the form of tillage on the soil. Adapted from the "The complexity of simple tillage systems" by Oswald et al., 2009, The Journal of Agricultural Science, 147(4), 399–410. Copyright 2009 by Cambridge University Press.

Peru is the center of origin of the potato *Solanum spp.*^6^, and its agricultural systems are recognized as a world patrimony^7^. Currently, Peruvian farmers continue to use traditional sowing systems^8–11^. Potato grows mainly between 3500 and 4300 masl, and for its sowing, Andean farmers developed three traditional tillage systems based on footplough: ‘chiwa’, ‘chacmeo’, and ‘barbecho’. ‘Chiwa’ is a minimal tillage system (MTS), in which ‘chakitaklla’ is commonly used (Fig. 1A). This pre-Inca instrument is used to posture the foot and is made of a 0.8–2.5 m long piece of wood with a 75–300 mm metal bar^9,10^. In this MTS, the ‘chakitaklla’ has been used on the pasture to perforate a hole where the potato seed is deposited at a depth of 0.1–0.2 m and is covered with the same soil. After three–four weeks, the soil near the planting area is turned or flipped to form ridges on the seeded tubers^10^. In the highland regions of the Peruvian Andes, the utilization of this technique remains a common practice, particularly for cultivation on mountain slopes; additionally, this method serves to mitigate the effects of nocturnal frosts, which are more pronounced in the plain terrains^10,11^. The ‘chacmeo’ is another type of MTS, wherein two clods of soil are organized to create a row or furrow for planting potatoes in between the clods; the ‘chakitaklla’ serves also as an integral instrument to this agricultural^10^. In turn, ‘barbecho’ is a full tillage system (FTS) that requires intensive soil preparation using the ‘yunta’ (for tilling the land with animal power support) or a tractor to turn, break and loosen the soil before sowing which facilitates the formation of furrows where the potato seeds are deposited^10^ (Fig. 1B).

Metagenomics has been used to understand the microbial communities, and is a high-throughput DNA sequencing technology used to study the genomic DNA contained in environmental samples, such as soil, sea environments, or freshwater habitats, to explore the taxonomic composition and potential uses of microorganisms that may benefit agriculture, bioremediation, and human health^2^. This sequencing technology has also been used to study intensive agriculture, land-use change, and agricultural soil in different countries, as well as to determine the effects of system tillage on soil microbial communities^12^. However, few studies have used this approach to study the soil microbiome in Peru, and no reports have focused on the influence of traditional tillage systems on soil microbiomes.

In this study, we utilized a shotgun metagenomic approach to study the bacterial community in Peruvian agricultural soil managed with traditional tillage systems such as ‘chiwa’ and ’barbecho’. We hypothesized that the use of these traditional tillage systems has an impact on the functional profile and taxonomic composition of soil microbiomes. Consequently, the objectives of this study were to characterize richness, diversity, evenness and taxonomic and functional composition of soil bacterial communities inhabiting two different Peruvian traditional potato tillage systems. Additionally, we determined potential bioindicators of soil management effects in these potato traditional tillage systems. For this, we selected the department of Huanuco in the Peruvian Central Andes because it is one of the major areas of potato production in Peru where farmers still use traditional potato sowing systems. These outcomes are the first starting point to understand the microbial communities and their ecology of Peruvian agricultural soil under traditional tillage systems.

## Results

### Physicochemical properties of soil samples

Soil physicochemical properties did not differ significantly between the traditional tillage systems (Table 1, *p*-value > 0.05). The soil samples were classified as sandy loam for MTS soils and sandy clay loam for FTS soils (Table S1). The PCA biplot of the physicochemical parameters revealed that soils under MTS exhibited a closer resemblance to soils under FTS (Fig. S1). Additionally, the PCA biplot indicated a positive relationship in soils under MTS with SOM and N, and soils under FTS exhibited a relationship with clay.

**Table 1:** Summary of physicochemical properties of soil samples from minimal tillage and full tillage systems. Values are presented as mean ± standard error (n = 2). Soil properties included soil pH (pH), soil organic matter content (SOM), available phosphorus (P), potassium (K), nitrogen (N), sand content (sand), clay content (clay), and silt content (silt). The p-values from the Wilcoxon rank-sum test are shown. Wilcoxon test was performed contrasting MTS *vs*. FTS samples across all physicochemical properties.

Physicochemical properties	Minimal tillage system (MTS)	Full tillage system (FTS)	*p*-value
pH	4.6 ± 0.2	4.2 ± 0.3	0.667
SOM (%)	18 ± 3.4	4.2 ± 2.2	0.333
P (ppm)	4.9 ± 0.7	5 ± 1.5	1
K (ppm)	113 ± 7	97.5 ± 2.5	0.333
N (%)	0.9 ± 0.17	0.2 ± 0.11	0.333
Sand (%)	72.2 ± 5.4	66.1 ± 9.3	0.667
Clay (%)	11.2 ± 0	21.2 ± 2	0.221
Silt (%)	16.6 ± 7.6	12.7 ± 7.3	0.667

### Traditional potato tillage system impact on ecological metrics of soil bacterial community

**Figure 2:**
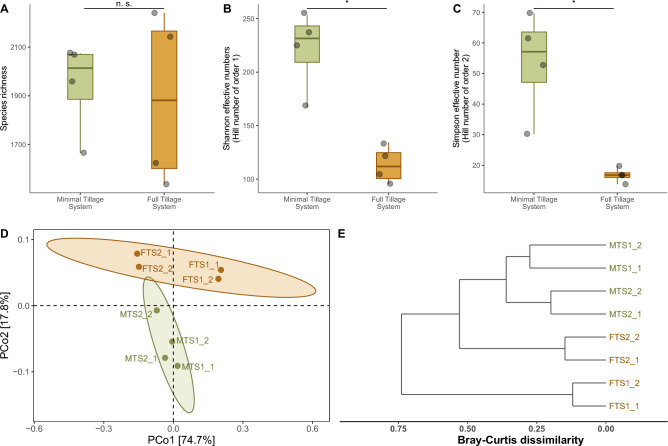
Potato soil bacterial microbiome alpha and beta diversity analyses. (**A**) Species richness. (**B**) Shannon effective numbers. (**C**) Simpson effective numbers. (**D**) Principal Coordinates Analysis using weighted UniFrac distances. (**E**) Dendrogram based on the Bray–Curtis dissimilarity * *p*-value < 0.05; n. s., not significant.

After quality filtering, we obtained an average of 9.3 million paired-end reads per soil sample (Table S2). Quality-filtered shotgun metagenomic reads were classified at rates of 5.9% and 6.8% for MTS and FTS, respectively, using Kraken2 (Table S2). These percentages encompass archaea, viruses, eukaryotes, and bacterial domains. Specifically, we focus solely on the bacteria domain, which constitutes an average of 3.3% and 4.4% for MTS and FTS, respectively. Taxonomic assignment recovered 4,488 bacterial species, representing 1,247 genera, 396 families, and 37 phyla (Table S3). Ecological metrics revealed no significant differences in richness for bacterial communities from MTS and FTS soils (Table S4, Fig. 2A). However, we observed significant differences in diversity and evenness indexes (Table S4, Fig. 2B,C). In beta diversity analysis, PCoA showed that MTS soils formed a clustered group, indicating similar composition, while FTS soil samples exhibited more heterogeneity (Fig. 2D); nevertheless, the application of a PERMANOVA revealed no evidence of differences between MTS and FTS, as indicated by the weighted UniFrac distances (R = 0.17792, *p*-value = 0.365) and the Bray–Curtis dissimilarity (R = 0.27515, *p*-value = 0.129). Additionally, the FT2 bacterial community was more similar to the MTS microbiome than FT1 (Fig. 2E).

### Bacterial community composition of soil under traditional tillage system

**Figure 3:**
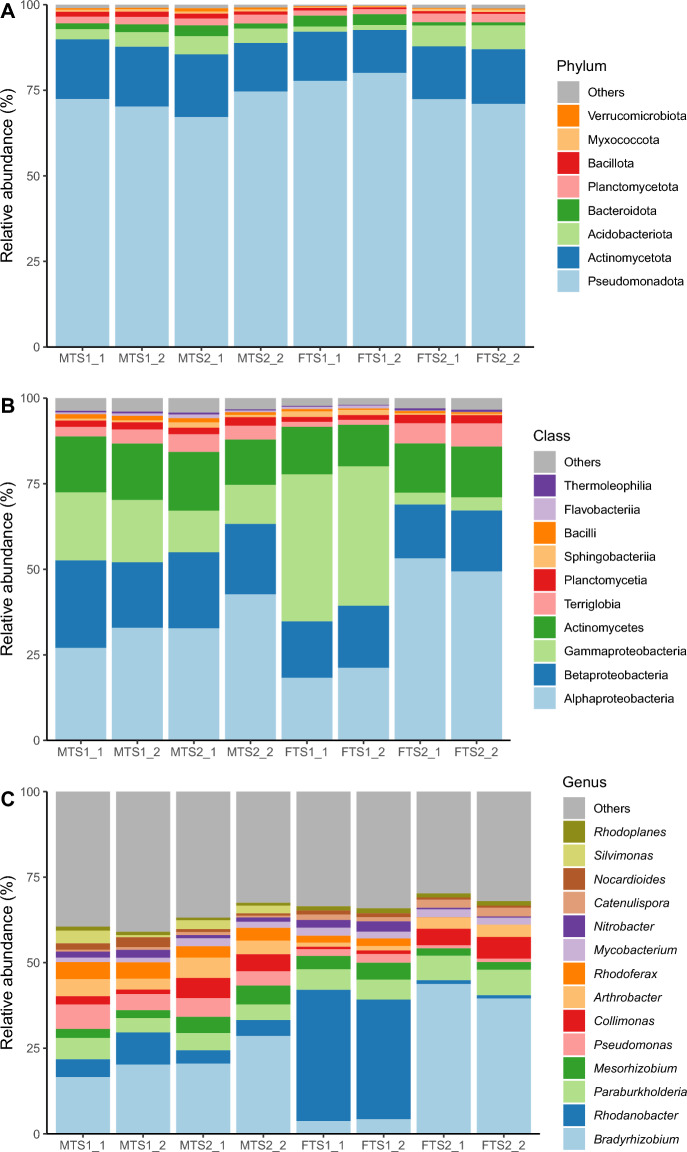
Bacterial taxonomic composition for traditional tillage systems (MTS: Minimal Tillage Systems, FTS: Full Tillage Systems) at the phylum (**A**), class (**B**), and genus (**C**) levels.

Pseudomonadota emerged as the predominant phylum across all the samples, accounting for 73.2% of the total bacterial composition. This phylum was followed by Actinomycetota (15.7%), Acidobacteriota (4.1%), Bacteroidota (2.1%), Planctomycetota (2.1%), and Bacillota (1.0%) (Table S5). In terms of class distribution, Alphaproteobacteria (34.6%), Betaproteobacteria (19.5%), Gammaproteobacteria (19.1%), Actinomycetes (14.8%), Terriglobia (4.0%), and Planctomycetia (2.0%) were the dominant taxonomical groups (Table S6). The prevailing families included Nitrobacteraceae (24.5%), Rhodanobacteraceae (13.0%), Burkholderiaceae (6.8%), Oxalobacteraceae (5.0%), Comamonadaceae (4.0%), and Acidobacteriaceae (3.7%) (Table S7). Among the genera, the most prevalent were *Bradyrhizobium* (22.2%), *Rhodanobacter* (12.3%), *Paraburkholderia* (5.8%), *Mesorhizobium* (3.5%), *Pseudomonas* (3.5%), *Collimonas* (3.4%), and *Arthrobacter* (3.4%) (Table S8). A comparison between the two tillage systems revealed that the phyla Acidobacteriota, Actinomycetota, Bacteroidota, and Bacillota (the latter four showing statistical significance), and along with Planctomycetota were more abundant in MTS soils, whereas only Pseudomonadota was more abundant in FTS soils (Fig. 3A, Table S9). At the class level, Actinomycetes, Bacilli, Betaproteobacteria, Flavobacteriia, Planctomycetia, Terriglobia, and Thermoleophilia were more abundant in MTS soils, whereas Alphaproteobacteria, Gammaproteobacteria, and Sphingobacteriia were more abundant in the FTS soils (Fig. 3B, Table S10). Among these classes, only Bacilli and Betaproteobacteria showed significant differences. At the genus level, *Mesorhizobium*, *Pseudomonas*, *Collimonas*, *Arthrobacter*, *Rhodoferax*, *Nitrobacter*, *Massilia*, *Nocardioides*, and *Silvimonas* were more abundant in the MTS soils. In contrast, *Bradyrhizobium*, *Rhodanobacter*, *Paraburkholderia*, *Mycobacterium*, and *Catenulispora* were more abundant in the FT soils (Fig. 3C and Table S11). Among these genera, *Pseudomonas*, *Rhodoferax*, *Catenulispora*, *Massilia*, and *Silvimonas* showed significant differences.

### Potato core microbiome after harvest under traditional tillage system

**Figure 4:**
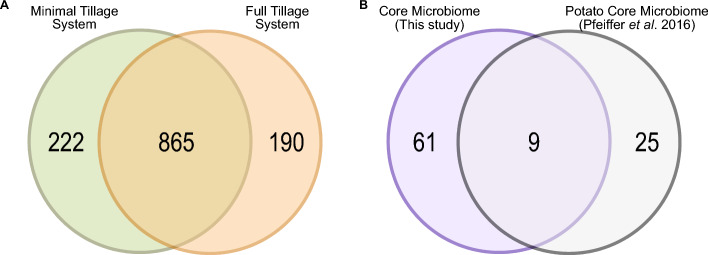
(**A**) Venn diagram of exclusive and shared bacterial species in traditional soil tillage systems. **(B)** Venn diagram comparing the core microbiome of this study with the potato core microbiome proposed by Pfeiffer et al. (2016).

A total of 835 genera (Fig. 4A, Table S12) and 2632 species (Table S13) were shared between the two tillage systems. We decided to focus our analysis on the genus level, considering only those with a minimum relative abundance of 0.1%. This approach was chosen to explore the core microbiome and achieve a more comprehensive understanding of the bacterial community. Giving these details, our potato core microbiome consisted of 70 genera (Table S14). This core microbiome encompasses eight phyla, with the majority of shared genera belonging to Pseudomonadota (32 genera), Actinomycetota (17 genera), and Acidobacteriota (8 genera). On average, this core represents 85% of the bacterial microbiome. When comparing to the Potato Core Microbiome proposed by Pfeiffer^13^, we identified nine genera in common: which are *Bradyrhizobium*, *Lysobacter*, *Massilia*, *Microbacterium*, *Nocardioides*, *Paenibacillus*, *Rhodoplanes*, *Streptomyces*, and *Variovorax* (Fig. 4B).

### Potential microbial indicators of soil management effects in potato traditional tillage systems

**Figure 5:**
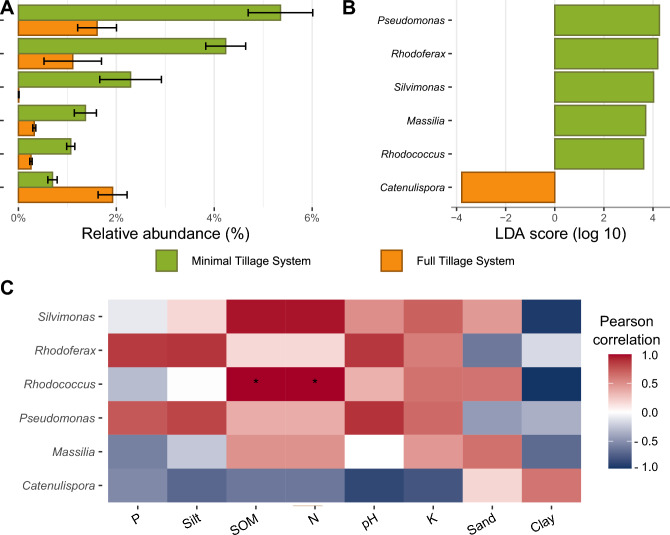
Bioindicators for soil under traditional tillage systems. (**A**) Relative abundance of bacterial genera for proposed bioindicators. (**B**) Differential abundance of bacterial bioindicators (based on LEfSe Analysis, *p*-value < 0.05, a minimum relative abundance > 1%, LDA threshold = 3.5). (**C**) Correlation between bacterial genera indicator and soil physicochemical properties. * *p*-value < 0.05.

We identified specific microbial genera that should be better investigated as early warning of MTS and FTS management effects. A total of 115 genera were found to be significant after the LefSe analysis (*p*-value < 0.05, Table S15); however, we filtered these genera to include only those with a relative abundance greater than 1% (Fig. 5A,B). In MTS soils, we detected five distinct genera, with four falling within the taxonomic classification of the Pseudomonadota phylum and one in the Actinomycetota phylum. These genera are *Pseudomonas*, *Rhodoferax*, *Silvimonas*, *Massilia, and Rhodococcus*. Notably, Pseudomonas, the most diverse of the identified genera, comprises 241 species, with *P. fluorescens* emerging as the most dominant among them. *Rhodoferax*, however, consists of nine species, with *R. sediminis* exhibiting the highest abundance within this genus. *Silvimonas* was represented by a solitary species. *Massilia* presented higher diversity, with 16 species, among which *M. putida* emerged as the predominant member. Whitin *Rhodococcus,* there are 34 species, with *R. globerulus* being the dominant species. Conversely, in FTS, we identified a single indicator genus, *Catenulispora*, which belongs to the Actinomycetota phylum, with *C. acidiphila* being the sole representative. We also established correlations between indicators and physicochemical parameters. The MTS indicators showed positive correlations with SOM, N, and K content; however, only *Rhodococcus* showed a significant relationship with these parameters (*p*-value < 0.05). In contrast, the FTS indicators exhibited a positive correlation with sand and clay (Fig. 5C).

### Soil bacterial functions in potato traditional tillage systems

**Figure 6:**
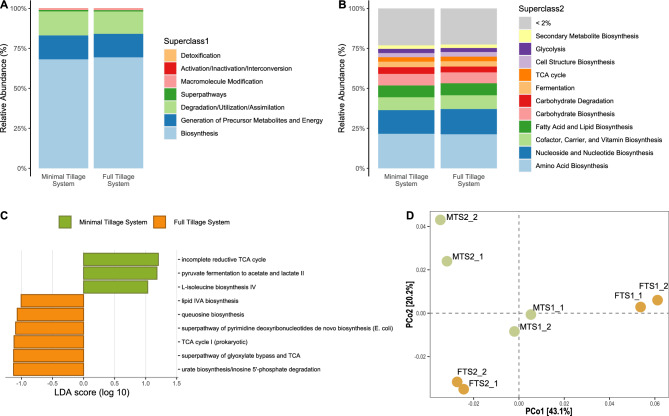
Functional profile for traditional tillage systems inferred by HUMAnN3. (**A**) Metabolic pathways grouped on superclass1 hierarchy. (**B**) Metabolic pathways grouped on superclass2 hierarchy. (**C**) LEfSe analysis to determine the most differentially abundant MetaCyc pathways. (**D**) Principal Coordinates Analysis based on MetaCyc pathways.

We recovered 435 metabolic pathways from our potato traditional tillage systems (Table S16), which were grouped into 43 higher-order functional MetaCyc ‘superclass 2’ (Fig. 6B), and seven ‘superclasses 1’ (Fig. 6A). At the ‘superclass 1’ hierarchy, both tillage systems displayed similar functional patterns, with biosynthesis (68.8%) as the main class pathway, followed by metabolism (14.8%) and degradation/utilization/assimilation (14.5%) (Fig. 6A, Table S17). At the ‘superclass 2’ level, almost two-thirds of the pathways were grouped in the biosynthesis of macromolecules (Amino Acids 21.5%; Nucleoside and Nucleotide 15.3%; Cofactor, Carrier, and Vitamin Biosynthesis 8.3%; Fatty Acid and Lipid 7.5%; and Carbohydrate 7.0%) (Fig. 6B, Table S18). LEfSe analysis identified 31 MetaCyc functional pathways with significant differential abundance among our potato traditional tillage systems (Table S19). We filtered those pathways with an LDA score above 1 and removed two MetaCyc pathways attributed to non-bacterial organisms. Within MTS, two of the three pathways belonged to the degradation/utilization/assimilation pathway, whereas for FTS, we recovered six pathways that were more diverse in their functionality (Fig. 6C). The pathway PWY-P42 ‘incomplete reductive TCA cycle’ was found to be the most remarkable function in MTS soils, which is connected to anaerobic conditions, and its main purpose is to fix carbon dioxide and produce organic molecules. The pathway PWY-5100 'pyruvate fermentation to acetate and lactate II' is also typically employed by anaerobic microorganisms to generate energy. The final significant pathway for MTS soils is PWY-5104 'L-isoleucine biosynthesis IV ’, which is crucial for the growth and survival of organisms, as it enables protein synthesis and various metabolic processes. In FTS soils, the pathway PWY-5695 ‘inosine 5'-phosphate degradation’ was the most significant function; it is involved in recycling nucleotide components. The second most important function of FTS soils is the ‘superpathway of glyoxylate bypass and TCA’ responsible for cellular energy metabolism and carbon utilization. The pathway ‘TCA cycle I (prokaryotic)’ is involved in aerobic respiration that generates energy and biosynthesis precursors. The pathway PWY0-166 ‘superpathway of pyrimidine deoxyribonucleotides de novo biosynthesis (*E. coli*)’ is involved in the synthesis of precursors for the synthesis of deoxyribonucleotides, which are essential for DNA replication and repair. The pathway PWY-6700 ‘queuosine biosynthesis I’ is important for the proper functioning of tRNAs, ensuring accurate translation during protein synthesis. The pathway PWY-8073 ‘lipid iva biosynthesis’ is responsible for the synthesis of lipopolysaccharides, which are essential components of the outer membrane in Gram-negative bacteria. Finally, PCoA of the metabolic pathways within each site revealed significant variability in their functional profiles (Fig. 6D). Although there was a degree of consistency in the metabolic pathways among replicates within the same site, these profiles exhibited substantial dissimilarity when comparing different sites. Notably, the metabolic pathways in MTS soils displayed a relatively close resemblance, whereas those in FTS soils exhibited a remarkable degree of heterogeneity.

## Discussion

In our research, we documented that in the central highlands of Peru (Huanuco) traditional tillage systems such as ‘chiwa’ and ‘barbecho’ are still used. These traditional tillage systems have been documented since the pre-Inca period^8,9^. It is important to document the continued use of these traditional tillage systems because they are part of the agricultural heritage shared between parents and children. In Peru, these systems have been developed for sowing crops, such as papa^10^, and mitigating the effects of climate instability, and improving seed stocks, and food reserves^14–16^. Minimal tillage is considered a key system in agricultural sustainability, because it reduces the negative effects of conventional agricultural systems and has beneficial effects on the physical, chemical, and biological properties of the soil^17^. This system also offers the advantage of lowering energy use and production costs^18^, impacting positively on carbon sequestration^19^, and improving soil texture and nutrient status^20^.

Although we did not find any statistically significant differences in the physicochemical properties of potato soils under traditional tillage systems, the literature indicates these differences. This could be attributed to the low sampling effort in our study. The fact that soils under MTS had a higher SOM content than those under FTS suggests a high potential for soil carbon storage^19^. This is also because these soils had no crop rotation during the last two years, and SOM accumulated. SOM comprises 45–60% of its mass as soil organic carbon (SOC), which is a principal source of energy for soil microbial communities^21,22^ and thus plays an important role in soil fertility^5,6^. Intensive tillage systems might affect SOC content in agricultural soil^22^. Therefore, promoting 'chiwa' tillage system plays a crucial role in the global carbon cycle, allowing the conservation of agroecosystem functions in response to climate change^16,23^. Additionally, the nitrogen content was higher in soils under ‘chiwa’ soils due to the possibility that residue decomposition could contribute significant amounts of N to the soil^20^. Furthermore, we registered that both traditional tillage systems had acidic soils which may reflect an inadequate use of fertilizers in the potato cultivation^24^, or the geological origins of these soils^25^, or soil leaching as a result of irrigation or rainfall^26^. On the other hand, the phosphorus content was similar between the systems; however, these soils can be classified as low-phosphorus soils, which is typical of acidic soils where the mineralization of phosphorus is limited^27^. Potassium content was higher in MTS soils as reported by previous studies^28,29^. Clay, silt, and sand are part of soil aggregates, which are important in the function of soil and are produced by mechanical or human activities in cultivated soil^30^. Some studies indicate that clay content is a predictor of SOM content^31^; however, other reports indicate that other physicochemical parameters are strongly related to SOM content^32^. We found that soils under MTS had lower clay content. This is in contrast with other studies that indicated that the clay percentage was higher in no-tillage systems^33^. However, this low clay content is relatively common in the central Andean highlands^10^. In addition, traditional tillage system of ‘chiwa’ may be considered within conservation agriculture, since soils undergo minimal mechanical alteration, leaving stumbles on the ground to protect the soil from sunlight and rainfall, enhance the biological activities of the soil, and reduce soil erosion^17^. Nevertheless, additional studies are needed to identify the factors that influence SOC, N, P, and K content and their relationship with traditional potato sowing systems in the central Andean highlands.

We observed that traditional soil tillage systems had no discernible impact on soil bacterial richness. Our findings align with previous studies that also reported no significant change in bacterial richness when comparing different tillage systems^28^. However, some studies found lower bacterial richness under no-tillage systems^34^, and others suggested higher bacterial richness^35^ when compared to conventional tillage. This observed variation may be attributed to factors such as functional redundancy among different bacterial species^36^. Additionally, bacterial richness could be influenced by factors such as the elevation of the sampling sites^37^, and soil acidity, which is a principal determinant of low bacterial richness^38^. Significantly greater diversity and evenness in bacteria communities were evident in MTS soils, likely resulting from the heterogeneity from higher SOM content and an undisturbed environment. Similar patterns have been observed in Chinese conventional tillage systems^33^, and in a global scale^39^. Regarding beta diversity, the Bray–Curtis dissimilarity index grouped the samples from MTS soils together, indicating a degree of similarity, while FTS soils displayed heterogeneity and did not cluster together. This divergence could be attributed to stochastic processes and a more dynamic environment within FTS soils. Furthermore, it is important to acknowledge that soil microbiomes can exhibit significant variation even over short distances^40^. The lack of clustering among similar tillage treatments in our samples may suggest a high level of heterogeneity Moreover, similar tillage treatments were not clustered together, which may indicate a high degree of heterogeneity in our samples, as observed within the agricultural soil microbiomes^41^.

Pseudomonadota, Actinomycetota, Acidobacteriodota, Planctomycetota, Bacteriodota, and Bacillota were the most abundant phyla in all soil samples under traditional tillage systems, which is similar to other reports in agricultural soils^33,34^. To understand the abundance of bacterial groups, we will use concepts based on the r and k strategist theory^42^. We found that soil under ‘chiwa’ systems had 18% SOM, while soil under ‘barbecho’ systems had 4%, with this characteristic of SOM we could assume that soils under ‘chiwa’ and ‘barbecho’ systems could be a putative environment for copiotrophic and oligotrophic microorganisms respectively. In the soils under ‘chiwa’ systems, we found microorganisms reported as copiotrophic such as Actinomycetota, Bacteriodota, Bacillota, and Pseudomonadota, of which only the Bacillota taxa showed significant differences. Actinomycetota, Bacteriodota, and Pseudomonadota have been reported in the rhizosphere of potato plants^13,42^; moreover, Pseudomonadota, and Bacteriodota have also been reported to have a positive relationship with residual potato yield^43^. At the class level, Bacilli, Betaproteobacteria, and Flavobacteria were more abundant in soils under MTS, with the first two being significant. Additionally, Bacilli, which are mainly obligate aerobes^44^, and Betaproteobacteria respond well to labile carbon sources in the soil^42^. These taxa are considered copiotrophic bacteria because of their fast growth, prosperity in nutrient-rich environments, lower carbon use efficiency^45^, and ability to decompose labile soils carbon reservoir^46^. In the soils under ‘barbecho’ systems, the phylum Pseudomonadota and the classes Alphaproteobacteria (copiotrophic) and Gammaproteobacteria (oligotrophic) were more abundant but lacked statistical significance. These findings indicate that although 'barbecho' systems exhibit lower levels of SOM, this reduction does not hinder the proliferation of copiotrophic microorganisms such as Pseudomonata, indicating carbon availability. Nevertheless, a more targeted and comprehensive study is required to draw a definitive conclusion regarding the dynamics of copiotrophic and oligotrophic microorganisms in both 'chiwa' and 'barbecho' systems.

A substantial number of genera and species were identified as shared components in both tillage systems. The core microbiome is intimately linked to networks that play a critical role in facilitating healthy plant microbiome interactions^1^. The higher number of shared genera and species observed in our study could be attributed to the evaluation of only one vegetation stage, in contrast to prior investigations on potatoes that considered different cultivars and encompassed four vegetation stages^13^. The potato rhizosphere microbiome is partially influenced by field site and climatic conditions^47,48^. Moreover, the effects of soil tillage systems on bacterial communities, as discussed in the previous section, highlight the role of soil properties in this context. In smaller proportions, the microbiome may be the result of opportunistic bacteria^13,41^ and cultivar-dependent bacteria^47^. Applying the criterion of focusing on bacterial genera with a minimum relative abundance of 0.1%^38^ revealed a core microbiome consisting of 70 genera. Compared with the Potato Core Microbiome proposed by Pfeiffer^13^, our analysis identified nine common genera. Among the genera shared in our study, *Bradyrhizobium* was included in the Potato Stable Core Microbiome^13^. *Bradyrhizobium* is known for its association with nitrogen fixation symbiosis in legumes^49^ and has been observed to be more abundant in potato fields with low disease incidence^50^. Another prominent member of our core microbiome is *Sphingomonas*, a genus also detected in fields with a low disease incidence^50^. *Sphingomonas* plays a dual role by promoting plant growth and enhancing plant resistance to pathogens^51^.

Metagenomics has been used to identify potential bioindicators of agricultural management effects on soils under different tillage systems^52,53^. *Pseudomonas*, *Rhodoferax*, *Silvimonas*, *Massilia, and Rhodococcus* were detected as bioindicator genera in soils under MTS. The first four genera belong to the phylum Pseudomonadota, which is recognized as a copiotrophic taxon, characterized by its ability to efficiently utilize available carbon sources^54^ and exhibit a positive increase in bacterial biomass when plant residues are present^55,56^. Additionally, Pseudomonadota plays an essential role in carbon, nitrogen, and sulfur cycles^12,57^, and is considered a potential bioindicator of agricultural management effects in no-till soils^53^. These characteristics could also explain the positive correlation with SOM, N, and K content of these four genera. Whereas *Cautenulispora*, a member of Actinomycetota phylum, was detected as a bioindicator of soils under FTS. Actinomycetota is considered an oligotrophic phylum and has been suggested as a microbial bioindicator of management effects in conventional tillage soils^53^. *Catelunispora* has been reported in acidic forest soils and paddy fields^58^; however, to our knowledge, this is the first report of these bacteria in soils associated with potato cultivation.

A comprehensive understanding of the metabolic pathways is essential for assessing the functional diversity inherent in traditional soil tillage systems^36^. Functionality in soil ecosystems is intricately linked to crop production and the overall health of these environments^2,3^. Both traditional tillage systems exhibit similar functional patterns at the 'superclass 1' level, with biosynthesis emerging as the predominant class pathway. Biosynthesis plays a central role in the functional profile of microorganisms in agricultural soils^59^. When comparing different functional pathways, MTS soils were associated with anaerobic conditions, with these pathways playing vital roles in processes such as carbon fixation and energy generation. These pathways are fundamental to primary metabolic processes, and their abundance can be attributed to SOM content, which is known to play a pivotal role in carbon metabolism^52^. In contrast, FTS soils exhibited pathways involved in nucleotide component recycling, along with key functions associated with energy metabolism, DNA replication, tRNA functionality, and lipid synthesis. Although there was some consistency among replicates within the same site, MTS soils demonstrated a relatively close resemblance, whereas FTS soils exhibited a remarkable degree of functional heterogeneity. Only 7% (31 out of 435) were differentially expressed between traditional tillage systems; this small number of features indicates functional redundancy^59^.

## Conclusions

Our findings revealed higher diversity and evenness for bacteria at the community level inhabiting soil under minimal tillage system in comparison with soil under full tillage system. However, traditional potato tillage systems had no discernible impact on soil bacterial richness. Similar soil bacterial community composition was shown for minimal tillage system, but it was heterogeneous for full tillage system, with copiotrophic and oligotrophic lifestyle microorganisms such as Pseudomonadota phylum, and Alphaproteobacteria and Gammaproteobacteria classes. This soil bacterial community composition under full tillage system may be attributed to stochastic processes related to activities of biosynthesis and degradation and a more dynamic environment within this tillage system. Moreover, this research opened the possibilities to explore microbial bioindicators for minimal and full tillage systems, which are related to healthy of soil, and they can be used to propose adequate tillage systems for the sowing of potatoes in Peru. These outcomes represent the first advance in the understanding of the ecology of soils in the Peruvian highlands under traditional tillage systems, which are important for the production systems of potatoes used by Peruvian farmers.

## Methods

### Agricultural fields description and soil sampling

The soil samples were collected from the Department of Huanuco in June 2017 (Fig. S2). Huanuco has an annual mean temperature ranging from 13.4 to 25.4 °C and an average precipitation of 38.1 mm per month. Peruvian farmers from two provinces of Huanuco used traditional tillage systems to sow potatoes: (1) ‘chiwa’ or MTS was identified in the province of Ambo, located between 3791 and 3881 masl, and this system was used for only sowing landraces potatoes; and (2) “barbecho'” or FTS was identified in the province of Yarowilca, located between 3648 and 3969 masl, and it was used for the sowing of improved potatoes (Fig. S2). Two agricultural fields were identified for each traditional tillage system. Soil samples were collected at harvest from a depth of 0–10 cm. For each field, nine random soil samples were collected. These samples were then mixed to obtain a composite soil sample, which was placed in a sterile Whirl–Pak bag for further analysis. Owing to the high level of similarity between the physicochemical parameters in the pre-analysis, we only examined one composite sample of soil per field for physicochemical characterization. For DNA sequencing, we considered two soil repetitions from each composite sample per field since our sequencing depth was required to be over 11 million reads and could have a good performance on species assignment; besides this depth is closer to the recommended sequencing depth^60^. Soil sampling was approved by the Servicio Nacional Forestal y de Fauna Silvestre (SERFOR) (permit number:151-2017-122 SERFOR/DGGSPFFS).

### Physicochemical analysis of soil samples

Physicochemical analysis was performed using the following parameters: pH, soil organic matter (SOM, %), nitrogen content (N, %), phosphorus content (P, ppm), potassium content (K, ppm), and texture (% of sand, clay, and silt). The pH determination was conducted using 10 g of soil. SOM was determined through the Walkey-Black method^61^. Nitrogen content was analyzed using the Kjeldahl method^62^. Phosphorus content was assessed using the Bray and Kurtz method^63^. Potassium content was determined using the spectrophotometric method. The soil texture was determined following the method outlined by Bouyoucos^64^. These analyses were carried out in the Laboratorio de Suelos of Estación Experimental Santa Ana of the Instituto Nacional de Innovación Agraria (INIA).

### DNA extraction and shotgun sequencing

Before DNA extraction, 500 g of the soil was dried at room temperature and sieved sequentially using 850, 500, and 212 µm meshes. One gram of sieved soil sample was used for DNA extraction using the PowerMax® Soil DNA Isolation Kit (MoBio Laboratories, Carlsbad, CA, USA), following the manufacturer’s protocol. The concentration and purity of the DNA were determined using an Epoch Biotek spectrophotometer, considering samples with absorbance values in the A260/280 and A260/230 ratio between 1.8 ~ 2.0 and 2.0 ~ 2.2, respectively. The integrity of the DNA was evaluated by electrophoresis on a 1% (w/v) agarose gel with 1X TBE buffer (Tris-HCl, boric acid, EDTA; pH 8.0) stained with ethidium bromide. For each well, 8 µL of loading buffer (bromophenol blue, xylene cyanol, orange G) was mixed with 2 µL of DNA and loaded into a horizontal electrophoretic chamber run in 1X TBE buffer at 100 V for 25 min. The results were recorded using a Bio-Rad transilluminator under UV light. Eight DNA sequencing libraries were prepared using Nextera XT DNA Sample Preparation Kit (Illumina, San Diego, CA, USA) according to the manufacturer's protocol using the Nextera index kit (Illumina, San Diego, CA, USA). Subsequent to indexing and clean-up processes, the libraries were pooled to an equimolar concentration of 1 nM and sequenced on the NextSeq 500 sequencing platform (Illumina, San Diego, CA, USA) to generate 2 × 150 bp paired-end reads. An average of 11.6 million raw paired-end reads were produced per soil sample; the minimum number of recorded reads for a single sample was 9.23 million, while the maximum reached 12.8 million paired-end reads (Table S2).

### Taxonomic assignation

The metagenomic data files were quality-filtered using fastp v0.23.2^65^ with the following parameters “-c -q 30 -n 0 -l 50”. Taxonomic assignment of the filtered reads was performed using Kraken2 v2.1.234^66^ using the following database “Standard plus protozoa, fungi & plant 20230605” with a 0.15 confidence threshold^67^. Abundances at taxonomic levels from species to phylum were reassessed using Bracken v2.8^68^.

### Functional profiling assignment

To recover the functional profile, the quality filtered, and concatenated shotgun metagenomic reads were classified to UniProt Reference Clusters 50 (UniRef50) with the ‘uniref50_201901b’ database^69^ gene families, pathway abundances were normalized to relative abundances, by-passed the taxonomic classifier option, then mapped gene families to MetaCyc reactions and metabolic pathways with the ‘full_mapping_v201901b’ database using HUMAnN v3.6.1^70^.

### Statistical analysis

All statistical analyses were performed in R software v4.2.2 using factoextra v1.0.7, phyloseq v1.40.0, ggplot2 v3.4.0, microeco v0.14.2, hillR v0.5.2, file2meco v0.5.0, and microbiome v1.18.0 packages. For physicochemical analysis, the mean and ± standard error for each parameter and tillage system were calculated, as well as a Principal Component Analysis (PCA) to assess the relationship between the physicochemical parameters and the soil traditional tillage system. For alpha and diversity analysis, a phyloseq object was built with the taxonomic assignment matrix obtained from Kraken2, and then filtered by the bacterial domain for further analysis. Richness, Shannon effective numbers, and Simpson effective numbers were calculated for each soil sample. For beta diversity, a weighted UniFrac distance matrix was employed in a principal coordinate analysis (PCoA), while a Bray–Curtis dissimilarity index was used to generate a dendrogram; subsequently, a permutational multivariate analysis of variance (PERMANOVA) was conducted using microeco, based on the adonis2 function from the vegan R package. The taxonomic composition at the phylum, class, and genus levels was assessed and plotted with relative abundance values. The core microbiome was defined by a minimum relative abundance threshold of 0.1% and an occurrence of 90% in all samples at the genus and species levels. The core microbiome was compared with the results of Pfeiffer et al.^13^ at the genus level and visualized using a Venn diagram. Soil tillage system bioindicators at the genus level were determined using linear discriminant analysis (LDA) effect size (LEfSe) analysis with a *p*-value of 0.05. The bacterial genera bioindicators were selected based on LDA for further analysis, and a correlation analysis between physicochemical parameters and bacterial bioindicators was performed. For functional analysis, HUMAnN3 metagenomic functional results were transformed into a microtable object. MetaCyc reactions were assessed at superclass1 and superclass2 categories of metabolic pathways, and then the functional different pathways were determined through a LEfSe analysis with a *p*-value of 0.05, and were filtered with an LDA score of 1.

### Supplementary Information

Supplementary Figures.

Supplementary Tables.

## Data Availability

The sequencing data are available at the NCBI Sequence Read Archive (SRA) under the Project Number PRJNA886760. Accession Numbers for MTS1 are SRR21793830, SRR21793833; MTS2, SRR21793827, SRR21793828; FTS1, SRR21793824, SRR21793825; and FTS2, SRR21793831, SRR21793832.
