# Supplementary material for: Traditional potato tillage systems in the Peruvian Andes impact bacterial diversity, evenness, community composition, and functions in soil microbiomes
Source: Sci Rep. 2024 Feb 17;14:3963. doi: 10.1038/s41598-024-54652-2 (PMC10874408; doi:10.1038/s41598-024-54652-2)

**Traditional potato tillage practices in the Peruvian Andes impact bacterial diversity, evenness, community composition and functions in soil microbiomes**

**Figure S1.** Principal Component Analysis (PCA) biplot of physicochemical properties of soil samples from minimal tillage systems (MTS) and full tillage systems (FTS). The biplot shows the PCA scores of the explanatory variables as vectors and individuals for each sampling site. Soil properties included soil pH (pH), soil organic matter (SOM) content, available phosphorus (P), potassium (K), nitrogen (N), sand content (sand), clay content (clay), and silt content (silt).


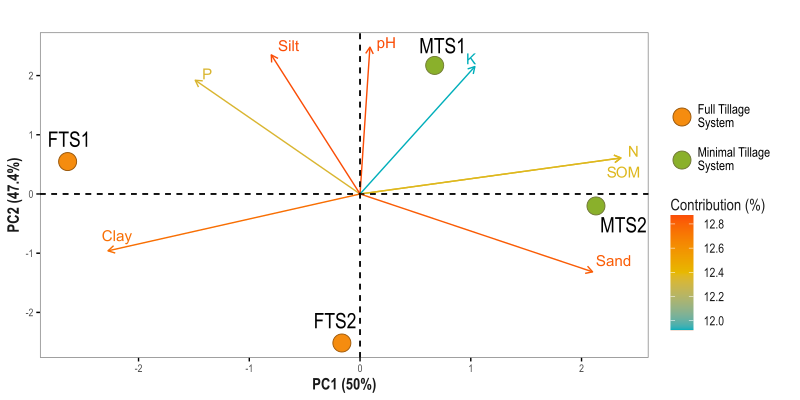


**Figure S2:** Sampling locations in the department of Huanuco, Peru. 'Chiwa' or minimal tillage systems (MTS1 and MTS2) sites were located in the Yarowilca province, while 'Barbecho' or full tillage systems (FTS1 and FTS2) sites were in the Ambo province. The base maps utilized were obtained from Natural Earth ([https://www.naturalearthdata.com](https://www.naturalearthdata.com/)), while river basin data was sourced from GeoIDEP ([http://mapas.geoidep.gob.pe](http://mapas.geoidep.gob.pe/)). Raster color scale adjustments and map composition were performed using QGIS v.3.24.2 (<https://qgis.org>), with final design accomplished in Inkscape v.1.0.2-2 (<https://inkscape.org>).


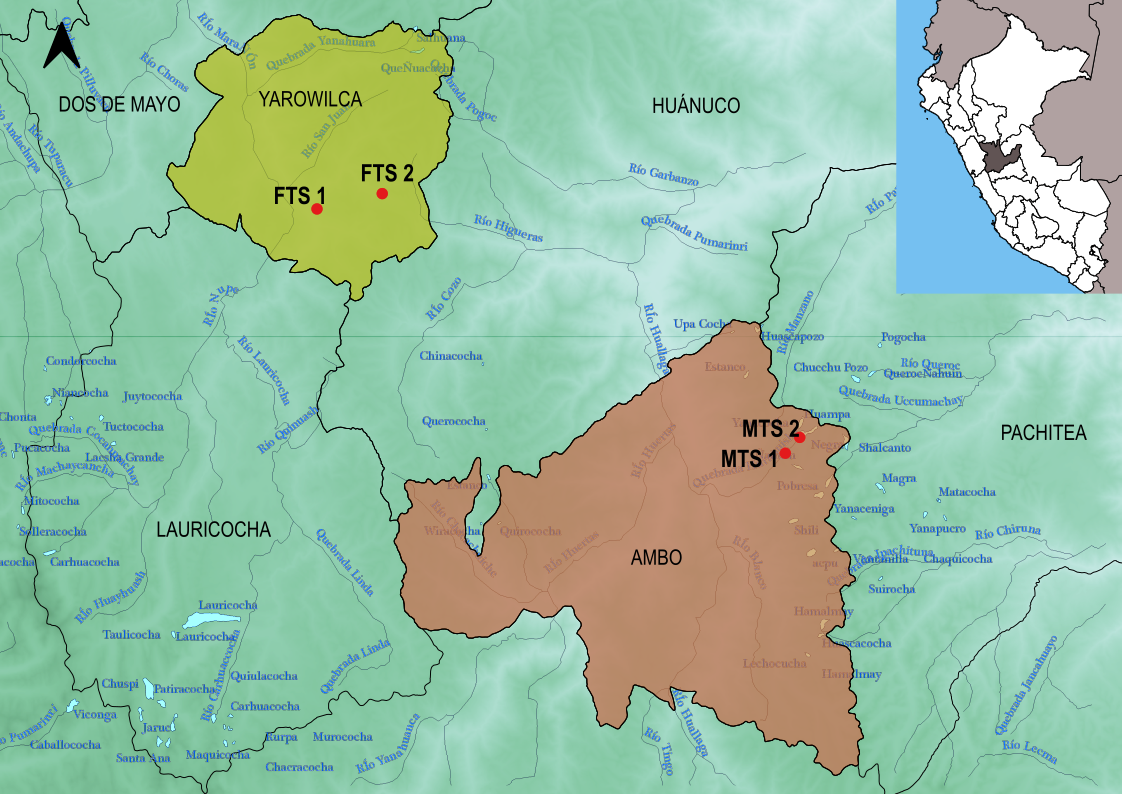

Supplement: Supplementary file 1 — Supplementary Figures. [file 41598_2024_54652_MOESM1_ESM.docx]
